# Autoantibodies to αS1-Casein Are Induced by Breast-Feeding

**DOI:** 10.1371/journal.pone.0032716

**Published:** 2012-04-04

**Authors:** Klaudia Petermann, Stefan Vordenbäumen, Ruth Maas, Achim Braukmann, Ellen Bleck, Thorsten Saenger, Matthias Schneider, Joachim Jose

**Affiliations:** 1 Institute of Pharmaceutical and Medicinal Chemistry, Westfälische Wilhelms-Universität Münster, Münster, Germany; 2 Department of Endocrinology, Diabetology and Rheumatology, Heinrich-Heine-University Düsseldorf, Düsseldorf, Germany; 3 Institute of Pharmaceutical and Medicinal Chemistry, Heinrich-Heine-Universität Düsseldorf, Düsseldorf, Germany; University of Hyderabad, India

## Abstract

**Background:**

The generation of antibodies is impaired in newborns due to an immature immune system and reduced exposure to pathogens due to maternally derived antibodies and placental functions. During nursing, the immune system of newborns is challenged with multiple milk-derived proteins. Amongst them, caseins are the main constituent. In particular, human αS1-casein (CSN1S1) was recently shown to possess immunomodulatory properties. We were thus interested to determine if auto-antibodies to CSN1S1 are induced by breast-feeding and may be sustained into adulthood.

**Methods:**

62 sera of healthy adult individuals who were (n = 37) or were not (n = 25) breast-fed against human CSN1S1 were investigated by a new SD (surface display)-ELISA. For cross-checking, these sera were tested for anti Epstein-Barr virus (EBV) antibodies by a commercial ELISA.

**Results:**

IgG-antibodies were predominantly detected in individuals who had been nursed. At a cut-off value of 0.4, the SD-ELISA identified individuals with a history of having been breast-fed with a sensitivity of 80% and a specificity of 92%. Under these conditions, 35 out of 37 sera from healthy donors, who where breast-fed, reacted positively but only 5 sera of the 25 donors who were not breast-fed. The duration of breast-feeding was of no consequence to the antibody reaction as some healthy donors were only short term breast-fed (5 days minimum until 6 weeks maximum), but exhibited significant serum reaction against human CSN1S1 nonetheless.

**Conclusion:**

We postulate that human CSN1S1 is an autoantigen. The antigenicity is orally determined, caused by breast-feeding, and sustained into adulthood.

## Introduction

The healthy human fetus is generally considered not to be significantly engaged in specific immunoglobulin production [1], [2]. Protection from pathogens *in utero* are conveyed by the placental barrier and transfer of protective antibodies of the mother [3], [4]. In contrast, infections *in utero* or immediately post partum as well as vaccinations can prompt specific antibody production against pathogens in neonates [1], [5], [6]. In the absence of foreign antigens in the fetus, self proteins may serve as an antigenic stimulus [7], creating antibodies that may be mono- or polyspecific and directed against self-tissue components [7], [8]. Although their precise role is currently unknown, it is speculated that these autoantibodies may confer protection against foreign pathogens [8] or help to survey the state of the individual's own cells [7], [9]. Illnesses induced by these early formed autoantibodies are only rarely observed [10]. Interestingly, these autoantibodies are not equally present in adults [7], [8], suggesting that autoreactive B-cells are mostly eliminated in the healthy maturing immune system.

Milk contains numerous proteins exposed to the immune system of neonates during nursing. Caseins are the main milk proteins of almost all mammalian species [11], [12]. They are a heterogeneous group of phosphoproteins, forming micelles with calcium phosphate and other components. The main biological function of caseins is to provide the progeny with a source of phosphate and calcium for the mineralization process of calcified tissues as well as amino acids [13]. In the course of natural evolution, some proteins are known to take up tasks besides their supposed initial functionality, a process known as protein promiscuity [14]. In recent years, evidence accumulated that caseins are such proteins: multiple immunomodulatory functions including modulations of the innate immune response of intestinal cells have been described. Recent research unveiled that human αS1-casein (CSN1S1) possesses immunomodulatory properties. Human CSN1S1 is expressed in monocytes and stimulates the expression of proinflammatory cytokines e.g. GM-CSF [15].

The current study aimed to investigate if a protein exposed to the immune system during nursing can generate a persistent antibody reaction and focused on the above described multifunctional, milk-derived protein CSN1S1. For this purpose, serum of 62 healthy volunteers who were or were not breast-fed was assessed for CSN1S1 antibodies using a SD-ELISA based on *E. coli* displaying the protein, similar to a strategy that was established before for human autoantigen Ro/SS-A [16]. It turned out that a history of having been breast-fed was strongly associated to an IgG-antibody reaction against CSN1S1, whereas a negative reaction stood into relation with formula feeding. (i.e. no exposure to human milk-proteins).

## Materials and Methods

### Human sera

62 human sera from healthy donors were obtained by puncture of the antecubital vein. Donors, who had been selected randomly, were free of medication and consisted of 43 female and 19 male persons of an age ranging from 22–73. All sera were stored at −20°C prior to the assay. All donors gave their full informed consent and the study was approved by the local ethics committee (Ethikkommission der Medizinischen Fakultät der Heinrich-Heine-Universität, Moorenstr. 5, D-40225 Düsseldorf).

### Materials

Goat anti-human IgG conjugated with horseradish peroxidase was obtained from Beckman Coulter (Krefeld, Germany), rabbit anti-human αS1-casein (CSN1S1) was obtained from ModiQuest (Nijmegen, The Netherlands), goat anti-rabbit IgG conjugated with horseradish peroxidase was obtained from Sigma-Aldrich (product number A0545 Munich, Germany) and goat anti-rabbit IgG conjugated with FITC was obtained from Bethly (Montgomery, USA). The restriction endonucleases were purchased from New England Biolabs (Ipswich, MA, USA). 3,3′,5,5′tetramethylbenzidine (TMB) was obtained from Sigma-Aldrich (Munich, Germany). Maxisorp® microplates were purchased from Nunc (Langenselbold, Germany).

### Bacterial strains and plasmids for CSN1S1 surface display

**Figure 1:**
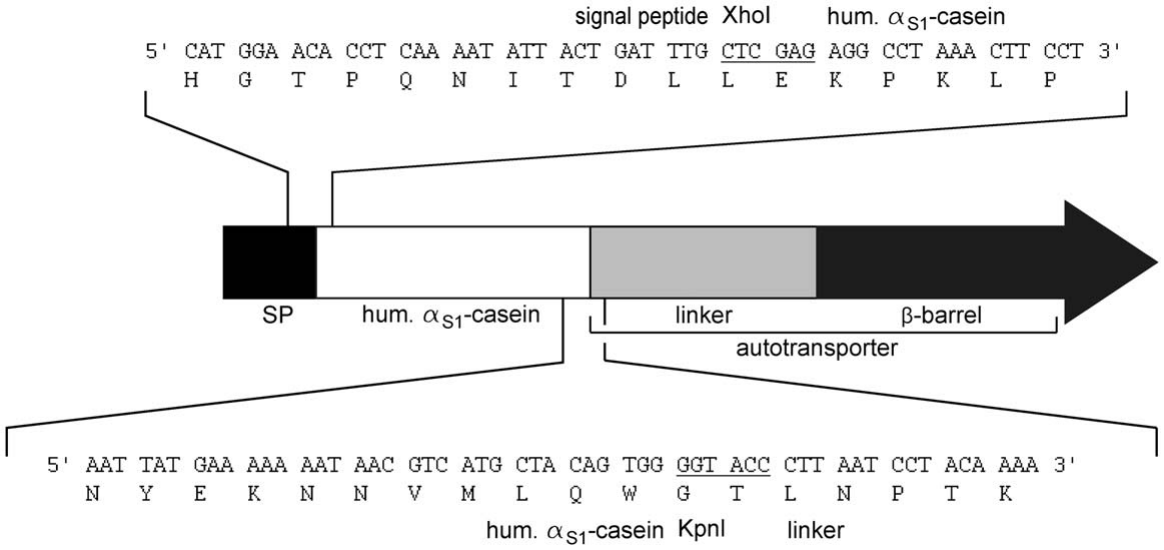
Structure of the CSN1S1 fusion protein for SD ELISA development. The environment of the fusion sites is given as sequences. The eight amino acids at the N-terminus and the two amino acids at the C-terminus that were added to CSN1S1 due to the cloning procedure are shown in italics. Restriction sites used for cloning are underlined. SP, signal peptide.

*E. coli* UT5600(DE3) [(F- ara14 leuB6 azi-6 lacY1 proC14 tsx-67 entA403 trpE38 rfbD1 rpsL109 xyl-5 mtl-1 thi1, ompT-fepC266)] [17] was used for the protein surface display. Plasmid pHaS1C2 [18] was used for amplification of human CSN1S1 gene without signal peptide by PCR. Plasmid pET Adx04, which encodes the AIDA-I autotransporter and Adx04 as passenger, was used for construction of the gene encoding the precursor protein needed for the surface display of CSN1S1 [19]. The CSN1S1 encoding DNA fragment amplified by the primers RM001 [5′-GCC TCG AGA GGC CTA AAC TTC CTC TTA G -3′] and RM002 [5′- CCG GTA CCC CAC TGT AGC ATG ACG -3′] was inserted by XhoI and KpnI restriction sites in plasmid pET Adx04 [19] and yielded plasmid pKP10 (Fig. 1). This resulted in an in frame fusion of the CSN1S1 encoding DNA region with the gene needed for surface display in *E. coli*. For pre-absorption of human sera, *E. coli* UT5600 (DE3) pET-SH3 [17] was used, expressing all the domains needed for autodisplay except CSN1S1. Bacteria were routinely grown at 37°C in Lysogeny-Broth (LB) containing 50 mg/L of ampicillin with ethylenediaminetetraacetate (EDTA, final concentration 10 µM) and β-mercaptoethanol (10 mM). SDS-PAGE, outer membrane protein preparation, surface detection of CSN1S1 and flow cytometer analysis was performed as described before [16].

### Indirect immunofluorescence

*E. coli* cells were grown as described above. After induction of protein expression, cells were labelled and fixed on a microscope slide, sealed with a cover slip. Analysis followed by a Zeiss® microscope (Axioskop 2 plus) using an extinction at 490 nm and emission at 515 nm.

### SD-ELISA

The principle of the SD ELISA is shown in Fig. S1. After induction of protein expression, *E. coli* cells were washed twice with PBS (pH 7.4) and applied to the wells of 96 well microplates (maxisorp®; Nunc, Langenselbold, Germany) in portions of 100 µl (OD_578_ = 0.5). After incubation overnight at 37°C, a blocking step followed with 150 µl PBS (pH 7.4)+10% FCS for 3 h at RT. Subsequently, incubation with human sera (100 µl; 1∶200) or – as positive control - with a polyclonal rabbit anti-CSN1S1 serum followed for 1 h at RT. Wells were washed 3× with PBS (pH 7.4)+0.1% Tween 20. As next step, 100 µl of goat anti-human IgG conjugated with horseradish peroxidase (HRP; 1∶10,000 in PBS with 10% FCS) was added. After 45 min at RT, wells were washed 3× with PBS (pH 7.4)+0.1% Tween 20 and 100 µl of 3,3′,5,5′tetramethylbenzidine (TMB) was added. Incubation in the dark at RT followed (25 min) and the reaction was stopped with 100 µl 2 M H_2_SO_4_. A_450_ was measured with a microplate reader (Berthold Technologies, Bad Wildbad, Germany).

### Preabsorption of sera

*E. coli* UT5600(DE3) pET-SH3 was grown overnight (37°C, 200 rpm) and expression of the control protein was induced by adding 1 mM IPTG for 60 min at 30°C and shaking (200 rpm). Cells were harvested, washed once in PBS (pH 7.4) and suspended to a final OD_578_ of 20 in PBS (pH 7.4). 145 µl of this solution was mixed with 5 µl human serum incubated for 5 hours (37°C, 800 rpm) using a thermo shaker (Labnet Vortemp 56, Oakham, UK). Subsequently, the supernatant was harvested, transferred to reaction tube with the pellet of 145 µl bacterial cell solution (OD = 20) and incubated for another 15 h (37°C, 800 rpm). Supernatant was harvested again and diluted with 850 µl PBS (pH 7.4) containing 10% FCS, resulting in 1∶200 final dilution of the serum applied finally to the SD-ELISA.

## Results

### Autodisplay of αS1-casein (CSN1S1)

For SD-ELISA (surface display ELISA) in order to detect an antibody reaction against CSN1S1, the human protein was displayed on the surface of *E. coli*. This was facilitated by Autodisplay, an efficient and convenient surface display system in *E. coli* [20], [21]. By similar means, an SD-ELISA for human Ro/SS-A autoantigen has previously been established: Validation of the assay on Ro/SS-A positive patients with systemic lupus erythematosus and healthy volunteers resulted in a sensitivity and selectivity that was at least identical, if not improved in comparison to commercially available standard ELISA [16]. The advantage of SD ELISA is that protein purification for coating the ELISA microplate with the antigen is not necessary (see Fig. S1: Schematic description of the SD-ELISA for CSN1S1 detection in human sera). The microplates used in an SD ELISA are simply coated by whole cells of *E. coli* displaying the target antigen. For Autodisplay of human CSN1S1, the corresponding gene was amplified by PCR and inserted into the plasmid pET Adx04 [19], resulting in plasmid pKP010 (Fig. 1). Plasmid pKP10 directed the expression of an artificial gene encoding a fusion protein comprising a signal peptide at the very N-terminus, CSN1S1, a linker domain and the β-barrel responsible for outer membrane translocation. Plasmid pKP010 was transformed into *E. coli* UT5600(DE3) and the expression of the CSN1S1 fusion protein was analyzed by SDS-PAGE. For this purpose, outer membrane proteins were prepared from *E. coli* UT5600(DE3) pKP010 cells after protein expression induced by addition of 1 mM IPTG. *E. coli* UT5600(DE3) cells without plasmid served as a negative control. IPTG addition resulted in a further protein band corresponding to the calculated size of the CSN1S1 fusion protein (approximately 70 kDa). A band of similar size was not detectable in control cells. This indicated that the CSN1S1 fusion protein was expressed in the outer membrane of *E. coli*.

To find out whether human CSN1S1 was indeed displayed at the cell surface, two different types of experiments were performed. One option to prove the surface display of a protein is to investigate its accessibility to proteases added from the exterior. Proteases like trypsin are too large to cross the outer membrane and only proteins displayed at the cell surface can be digested. As seen in figure 2.1, lane 2, the 70 kDa band representing the CSN1S1 fusion protein completely disappeared after incubation with trypsin. In both samples natural outer membrane proteins Omp F/C and OmpA remained unaffected. Because OmpA has a periplasmic extension directed towards the inner side of the outer membrane, this indicated that the cell envelope remained intact during trypsin treatment.

**Figure 2:**
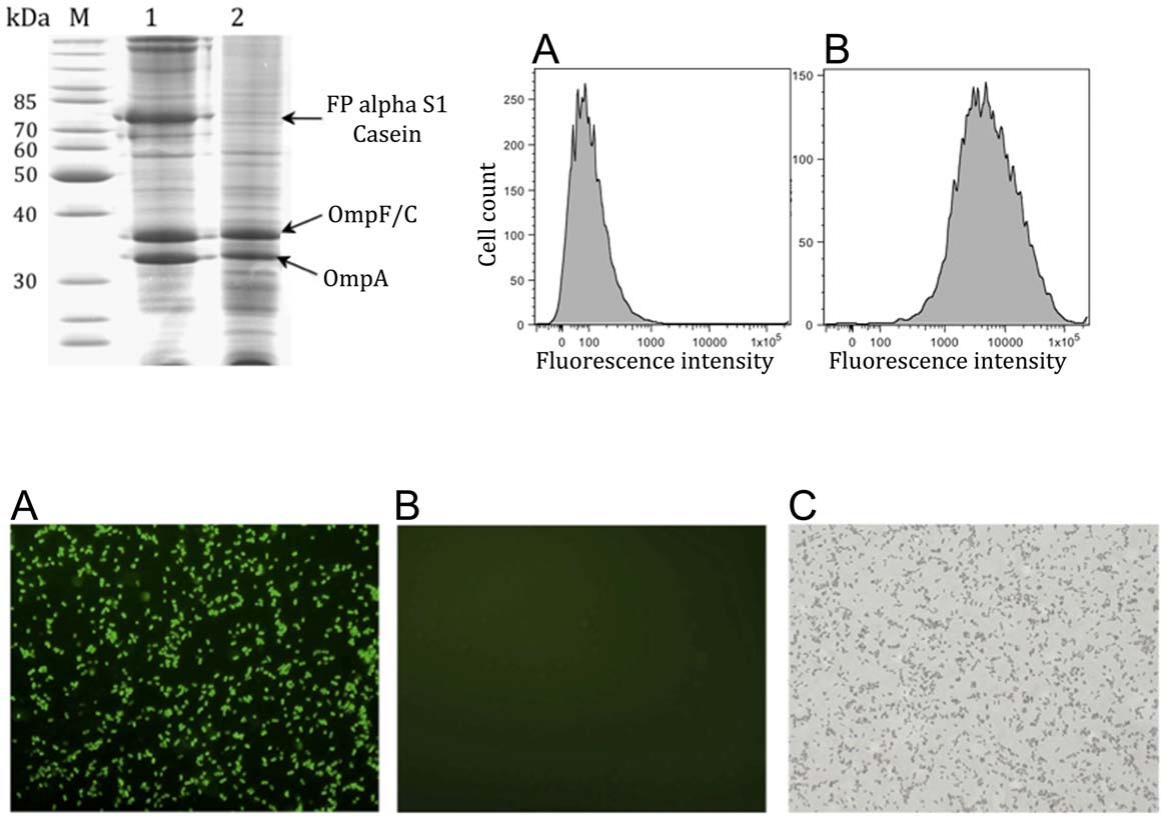
Surface accessibility of human CSN1S1 on *E. coli*. 2.1) SDS-PAGE of outer membrane protein preparations from *E. coli* UT5600(DE3) pKP010 which were incubated without trypsin (lane 1) and cells which were treated with trypsin (lane 2). OmpF/C and OmpA are natural outer membrane proteins of *E. coli*, and can be used as reporters for successful outer membrane protein preparations. 2.2) Flow cytometer analysis of *E. coli* cells displaying αS1-casein. (A) *E. coli* UT5600(DE3) pKP010 were incubated with a polyclonal rabbit antiserum against CSN1S1 casein and subsequently with a secondary antibody conjugated with FITC. (B) The mean fluorescence of *E. coli* UT5600(DE3) pKP010 was 10,071. The value of the fluorescence for *E. coli* UT5600(DE3) cells without a plasmid, used as a negative control, was 464. 2.3) Fluorescence microscopy of both cell types. The cells were treated identically as described for flow cytometer analysis (2.2). (A) *E. coli* UT5600(DE3) pKP010 ; (B) *E. coli* UT5600(DE3) as negative control; (C) *E. coli* UT5600(DE3) as negative control, transmission light control.

To support this finding, *E. coli* cells displaying CSN1S1 were subjected to indirect immunofluorescence using a polyclonal rabbit serum against CSN1S1. Antibodies like proteases are too large to be able to cross the outer membrane. As a consequence, in case the rabbit antiserum detects the CSN1S1 domain, it must be displayed at the cell surface. *E. coli* UT5600(DE3) pKP010 cells and *E. coli* UT5600(DE3) cells as control were incubated with rabbit anti-human CSN1S1 serum for 30 min at room temperature. After repeated washing, incubation with a goat anti-rabbit IgG conjugated to FITC followed and whole cell fluorescence was analyzed by flow cytometry. As seen in Fig. 2.2, the mean fluorescence (mF) of cells expressing the CSN1S1 fusion protein (10,071) was 22 times as high as that of control cells (464), thus indicating that the CSN1S1 domain was indeed accessible at the cell surface. In addition, cells displaying human CSN1S1 were labelled accordingly, but analyzed by immunofluorescence microscopy. As shown in Fig. 2.3, again only cells displaying CSN1S1 on the surface exhibited a green fluorescence, caused by binding of the rabbit anti-CSN1S1 antibodies and a FITC labelled secondary anti-rabbit IgG antibody.

### SD-ELISA with sera of 62 healthy volunteers

**Figure 3:**
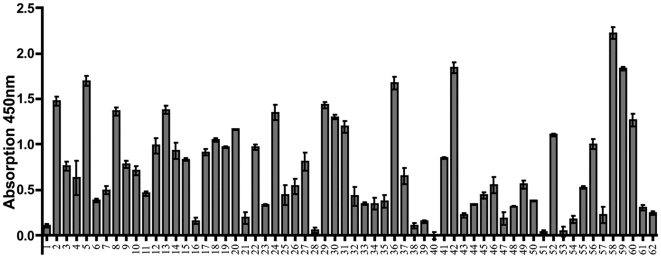
Antibody reaction against human CSN1S1 in human sera. 62 sera of healthy volunteers were tested for antibodies against CSN1S1 with the SD-ELISA. Every serum was measured three times independently, the average was calculated and is shown as a column. SD of each mean is given as a line.

For each patient serum to be tested, three wells of a 96 well plate were coated with *E. coli* UT5600(DE3) pKP010 and additional three wells were coated with *E. coli* UT5600(DE3) as a control. In order to avoid effects caused by the host *E. coli*, and therefore being independent of CSN1S1, the absorption values obtained from the control *E. coli* UT5600(DE3) cells were subtracted from the values obtained with *E. coli* displaying CSN1S1, as it has been done before in the Ro/SS-A SD-ELISA [16]. The difference in the mean of the absorption values measured for each cell type were supposed to represent the serum reaction against mere CSN1S1. As consequence, the absorption values obtained for each serum were averaged and standard deviations were calculated. Finally, 62 sera obtained from healthy volunteers were tested by using this protocol. As summarized in Fig. 3, a remarkable antibody reaction against CSN1S1 was detectable in 40 out of the 62 sera analyzed.

**Figure 4:**
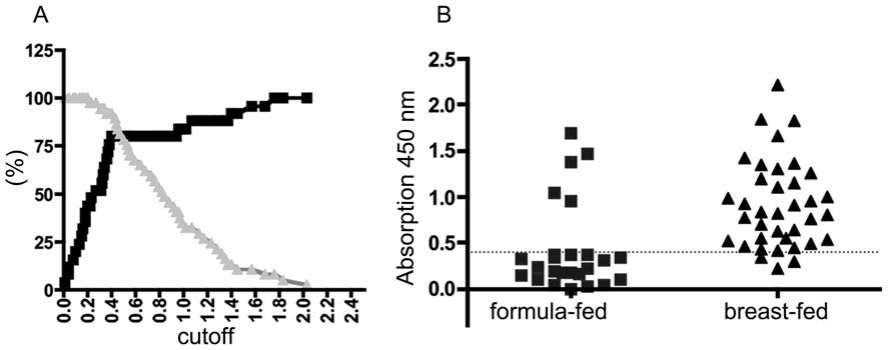
Analyzing the serum reaction against human CSN1S1 with respect to formula-fed and breast-fed test persons. 4.1) For each cut-off value, sensitivity and specificity were calculated to choose the optimal cut-off value for the assay which was 0.4. sensitivity; ▪ specificity ▴ 4.2) The 62 probands were divided into two collectives (formula-fed and breast-fed). 25 of the 62 volunteers were formula-fed and 37 were breast-fed. Only 5 sera of the formula-fed collective have an absorption value over 0.4 and 2 sera of the breast-fed collective have an absorption value under 0.4. (dotted line = cut-off value).

**Figure 5:**
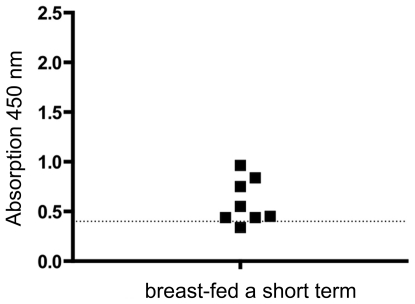
Serum reaction against human CSN1S1 in probands who were breast-fed for a short period of time. Eight probands who were breast fed for a short period of time (5 d–42 d), showed an increased antibody reaction against CSN1S1 in comparison to control persons, who were not breast-fed. (dotted line = cut-off value from Fig. 4).

To analyze the hypothesis that a history of breast-feeding was associated with the development of anti-CSN1S1 antibodies, healthy donors were divided into two groups: a breast-fed and a formula-fed consortium. This resulted in two clear cut and significantly different populations and a cut-off analysis could be performed. For each possible cut-off value, the sensitivity and specificity was calculated by GraphPad Prism® [22], [23]. These values were plotted against the different cut-off values (Fig. 4A). In the SD ELISA presented here, using cells of *E. coli* displaying human CSN1S1 as an antigen source, a cut-off value of 0.4 was defined (Fig. 4A) to distinguish positive from negative reactions. At this value, a maximum balance between sensitivity and specificity was obtained. As a consequence, sensitivity had a value of 80%, whereas specificity was 92%. In this case, the sera of the breast-fed group had absorption values clearly over 0.5. As shown in Fig. 4B, under these conditions, only 5 sera of the 25 donors, who were formula-fed, showed an antibody reaction against CSN1S1 beyond the cut off, whereas 35 sera of the 37 donors, who where breast-fed, reacted positive. There was no significant difference in this antibody reaction with relation to sex or age of the healthy donors analyzed (see Fig. S2: Comparing the serum reaction against CSN1S1 of female and male test persons with respect to being breast-fed as a neonates). Eight donors of the breast-fed group were only breast-fed for a short term (from 5 days until 6 weeks). After this period of time, they were formula-fed. As shown in Fig. 5, the limited duration of the breast-feeding period had no influence on the strength of the serum reaction. This emphasized the importance of breast-feeding as such for stimulating a serum reaction against CSN1S1.

**Figure 6:**
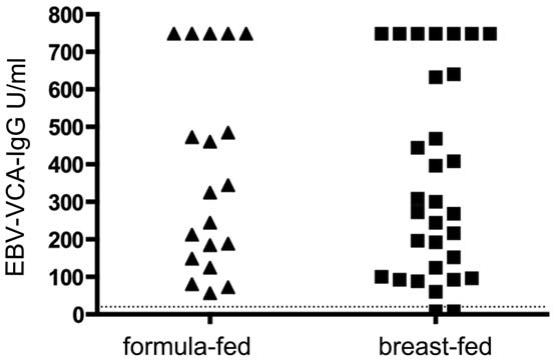
Serum reaction against EBV-VCA in formula-fed and breast-fed probands analyzed as a control. A commercially available ELISA for antibodies against EBV-VCA was used as described in material and methods.

For cross checking, all sera were also investigated for antibodies against Epstein-Barr virus (EBV) by a commercial ELISA, in order to exclude that breast-feeding in general results in an enhanced immune reaction. EBV was used because it is known, that almost 95% of all adult humans possess antibodies against the virus. Accordingly, it was to expect that the majority of sera would react on EBV. Our aim was to find out whether this reaction is enhanced in persons being breast-fed, or whether there is an equal, but random distribution in strength between sera from breast-fed and non–breast fed persons. As can be seen in Fig. 6, there was no significant difference in the strength of the serum reaction against EBV between the two consortia, breast-fed and non-breast fed (*P = *0.213). The absorption values of both groups spread equally between 60 and >750 U/ml. According to the manufacturers recommendation, the cut-off value is 10 U/ml for this ELISA test (against EBV virus capsid antigen, VCA). Accordingly, 60 out of the 62 sera analyzed showed a serum reaction against EBV-VCA. This represented 96% of all probands and corresponded to the values reported before [24]. The two persons whose sera were negative belonged to the group of breast-fed, a minor but further hint, that breast-feeding does not lead to an enhanced immune reaction in general.

Although medical records of all probands were negative for cow milk allergy and there is only weak sequence homology between human and bovine CSN1S1, with the exception of the first 20 amino acids, which belong to the signal peptide and which is cleaved off at position 15/16 between A and R during transport into the endoplasmatic reticulum (Fig. S3), it is important to experimentally verify, that the detected IgG mediated response to human CSN1S1 is not due to a cross-reactivity against the bovine milk protein, to which all probands are likely to be exposed later in life for most of their entire life span. In order to exclude such a contribution of subsequent exposure to bovine CSN1S1 in the development and maintenance of anti-human CSN1S1 antibodies, a similar SD-ELISA as described for human CSN1S1 was developed for the bovine protein. For this purpose the bovine CSN1S1 encoding DNA fragment was synthesized according to the available sequence information in the Genbank database (UniProt: P02662.2) with lateral restriction sites XhoI and KpnI and inserted in the Autodisplay expression vector as described for the human antigen (Fig. 1). Expression of the resulting fusion protein was monitored by SDS-PAGE and surface display of the bovine CSN1S1 domain was verified by a protease accessibility assay and by indirect immunofluorescence. Subsequently the cells displaying the bovine protein were used to analyze the serum reaction of the probands against this antigen at the identical conditions as described for the SD ELISA against human CSN1S1. With the exception of two sera, corresponding to sample no. 39 and sample no. 53 according to Fig. 3, none of the sera analyzed exhibited a reaction against bovine CSN1S1. Both samples no. 39 and no. 53 belonged to the group of formula-fed persons, who turned out to be negative towards a reaction against human CSN1S1. The reaction of the two sera was only weak (0.076 for no. 39 and 0.362 for 53) and below the cut off value for a positive reaction as determined for the human antigen. Hence, there is no correlation between the reaction against human CSN1S1 and bovine CSN1S1 in the analyzed human sera. These data suggest that cross-reactivity of human and bovine CSN1S1 antibodies can be excluded to be responsible for the observed effects.

**Figure 7:**
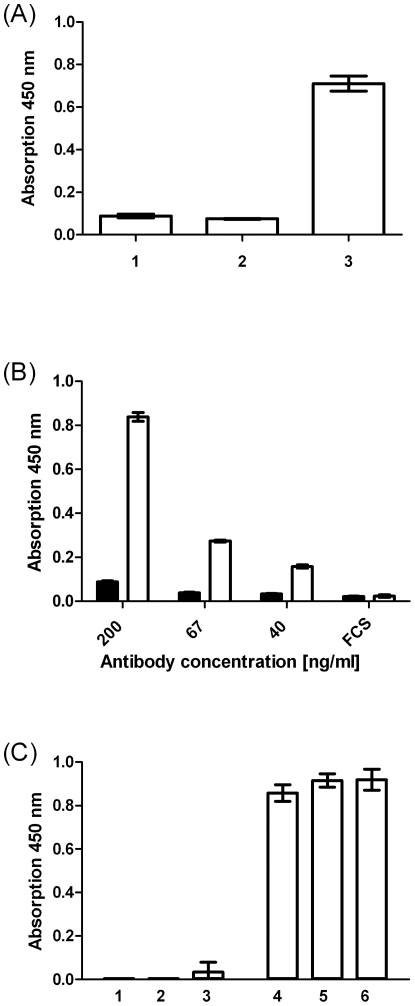
Antibody reaction against bovine αS1-casein. (A) Antibody reaction against *E. coli* UT5600(DE3) pSH3 (control, lane1), *E. coli* UT5600(DE3) displaying human CSN1S1 (lane 2) and E. coli UT5600(DE3) displaying bovine CSN1S1 (lane3) measured by SD-ELISA with a rabbit polyclonal anti- bovine CSN1S1 antibody. For detection a goat HRP conjugated anti rabbit antibody was used according to the conditions described for Fig. 3. (B) The antibody reaction against *E. coli* UT5600(DE3) pSH3 (control, black columns) and *E. coli* UT5600(DE3) displaying bovine CSN1S1 (white columns) were analyzed at different concentrations of the rabbit polyclonal anti-bovine CSN1S1 antiserum or in PBS with 3% FCS as negative control. (C) The SD ELISA against bovine CSN1S1 was repeated three times independently in triplicates at the highest antibody concentration applied (200 ng/ml) with *E. coli* UT5600(DE3)SH3 (control, lanes 1–3) and *E. coli* UT5600(DE3) displaying bovine CSN1S1 (lane4–6) in order to test the reproducibility.

For cross checking and in order to elucidate whether the SD ELISA against bovine CSN1S1 can yield higher absorption values in principle, a commercially available polyclonal rabbit antiserum directed against bovine CSN1S1 was applied with a secondary goat-anti rabbit IgG coupled with horseradish peroxidase. As can be seen in Fig. 7A, this resulted in an absorption of beyond 0.7 when cells displaying bovine CSN1S1 were used to coat the microplate well. In case controls cells of *E. coli*, or cells of *E. coli* displaying human CSN1S1 were used, the absorption values obtained were below 0.1. This again indicates that there is no cross reactivity between human and bovine CSN1S1 and emphasizes the negative results obtained with the human sera in the SD ELISA with bovine CSN1S1. The reaction obtained with the commercially available rabbit antiserum in the SD ELISA against bovine CSN1S1 was concentration dependant (Fig. 7B) and exhibited high reproducibility (Fig. 7C).

**Figure 8:**
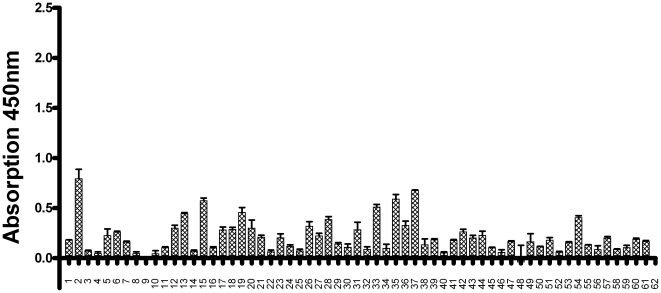
IgM reaction against human CSN1S1 in human sera. 61 of the 62 sera shown in Fig. 3 with the IgG reaction on human CSN1S1 were analyzed for an IgM mediated serum response on the same antigen by SD-ELISA. Each serum was tested three times, the mean was calculated and shown as columns. SD of each mean is given as a line.

**Figure 9:**
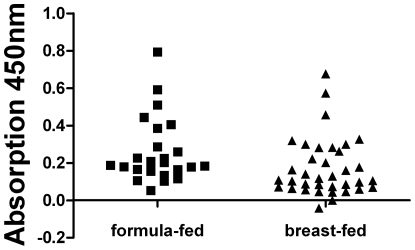
Analyzing the IgM serum reaction against human CSN1S1 with respect to formula-fed and breast-fed test persons. 61 of the 62 sera shown in Fig. 3 were divided into two consortia, a formula-fed and breast-fed, as it is shown for the IgG mediated response in Fig. 4. The formula-fed consortium comprises the sera of 25 volunteers, whereas within the breast-fed consortium 36 sera were analyzed.

Although the antigenic stimulus from breast-feeding during infancy would be expected to result in an IgG response rather than an IgM response, a surface display ELISA for anti human CSN1S1-IgM was developed in order to clarify if the serum response induced by breast feeding is effecting IgG exclusively or may also influences the IgM repertoire. The assay was performed under identical conditions, but instead of goat anti-human IgG conjugated with horseradish peroxidise, a goat anti-human IgM conjugated with horseradish peroxidise was employed. As can be seen in Fig. 8, which gives the difference in absorption between cells displaying human CSN1S1 and the absorption values obtained with control *E. coli* for each serum, the IgM mediated response was much lower than the response mediated by IgGs. Only insufficient amounts of serum no. 62 were available, and therefore excluded from this part of the investigation. The mean value of the reaction against control cells was 0.810 in case of the IgG dependant SD ELISA, while the corresponding value for the IgM dependant SD ELISA was 0.328, which appears to reflect the generally lower presence of IgM antibodies in comparison to IgG antibodies in human sera. Nevertheless, there were a few sera, that exhibited a difference in absorption beyond 0.4, the cut-off value determined in the IgG SD ELISA. However, there were no differences in IgM-responses between the breast-fed and formula-fed group. (Fig. 9). The mean value in case of the breast fed consortium was 0.174, whereas the mean value obtained with the formula fed consortium was 0.259. According to a one way analysis of variance (ANOVA) with subsequent Sheffé-test using WinStat. Version 2007.1, the mean values are not significantly different (*P* = 0.05).

## Discussion

In the present study, we analyzed whether there is a serum reaction against human αS1-casein (CSN1S1) in humans. Moreover, because CSN1S1 is a major component of breast milk, we investigated whether there is a difference in serum reaction concerning breast-feeding. 62 sera were analyzed with an SD ELISA as described before for Ro/SS-A [16]. At first sight, it clearly turned out that there is a significant reaction against CSN1S1 in human sera. The healthy donors were divided into two groups, a breast-fed (37 donors) and a formula-fed group (25 donors). Examining single absorption values revealed an average value of 0.432+/−SD for the formula-fed group and 0.894+/−SD for the breast-fed group. This difference was statistically significant in Mann-Whitney U-Test *(P = *0.002). Interestingly, it appeared that the length of breast-feeding has no influence on the antibody reaction. The serum of a person who stated to have been breast-fed for only one week exhibited the highest serum reaction against CSN1S1 among the short time breast-fed persons (Fig. 5). This is notable because the immune system of neonates is assumed to be incompletely developed [25], [26]. During pregnancy, the fetus obtains maternal IgGs *via* the placenta. As we analyzed sera of adults, this cannot account for the serum reactions observed. It rather appears that this immune response was acquired during breast-feeding and it cannot be excluded that this has happened during the earliest days of infancy.

There are reports suggesting that breast-feeding leads to an improved development of the immune system in infants [27], [28], [29]. For example, breast-fed children have a higher serum antibody-titer against *Haemophilus influenzae* type b [29] or diphtheria toxin [28] after vaccination. Thus, we had to exclude that breast-feeding leads to a stronger immune response in general, thereby accounting for higher CSN1S1-Ab titers in breast-fed individuals. This could be excluded by the example of equal serum reaction against EBV-VCA in both groups. EBV was used because nearly 95% of the adult population has antibodies against EBV-VCA, but in most if not all cases due to a contact in later life time. This notion is not necessarily contradicted by the above mentioned findings of higher antibody-titers of breast-fed children after *Haemophilus influenzae* type b or diphtheria toxin immunization. Indeed, based on the present results, one could raise the hypothesis that breast-feeding enhances the immune reaction of infants or new born, but this effect is lost or overlaid at later life time, a hypothesis of course, that needs further investigation.

At this point the question arises whether the enhanced serum reaction against CSN1S1 may be related to the wide-spread cow milk allergy (CMA). Bovine CSN1S1 is the main allergen in CMA, but in over 60% of all cases, this reaction is mediated by IgE antibodies [30]. In the present investigation, the serum reaction can clearly be assigned to IgGs. In addition to the IgE mediated immune reaction, 89% of all CMA patients exhibit an IgG mediated immune reaction against bovine CSN1S1. The domain targeted by these IgG antibodies was determined and found to be located between amino acids 15 to 36 of bovine CSN1S1 [31]. For this domain of bovine CSN1S1, however, there is no homologous domain existing in human CSN1S1 and therefore, a cross reactivity of IgG antibodies directed against bovine CSN1S1 with human CSN1S1 can be excluded (see Fig. S3: Amino acid sequence alignment of bovine and human αS1-casein (CSN1S1) using the Smith and Waterman algorithm). Finally, none of the persons whose sera were analyzed on an immune reaction against human CSN1S1 in the present study, reported averse reactions to cow-milk, suggesting CMA. In accordance with this, cross-reactivity of human CSN1S1 antibodies to bovine CSN1S1 could be excluded in the cohort by a novel SD ELISA for the bovine molecule.

It is notable that the strong IgG mediated immune reaction against human CSN1S1 in the sera of adults is obviously caused by breast-feeding, or in other words, by an oral application of the antigen during infancy. Well-known examples of oral vaccination in humans include immunization against polio and rotavirus. But in both cases, the immune response is raised against an – from an evolutionary point of view - old human pathogen, and it appears reasonable that any contact, even oral application, leads to a strong and sustaining immune response. Oral immunization is used in chicken for subsequent harvesting of antigen specific polyclonal antibodies from egg yolk. But in this case again, the antigens applied are no self-proteins but foreign antigens [32]. This is also true for the striking increased amount of IgG antibodies against food antigens in the jejunal perfusion fluid of rheumatoid arthritis patients when compared to healthy control persons [33]. To our knowledge, the data presented here for human CSN1S1 are the first example that oral application of a self-protein in earliest childhood leads to a strong IgG mediated immune response that is sustained into adulthood. While breast-fed induced reaction against CSN1S1 is clearly based on an IgG rather than an IgM response, we strikingly detected anti human CSN1S1-IgM antibodies (albeit in low concentrations) in both nursed and formula-fed individuals without any difference. This might suggest a more recent antigenic stimulus. It has recently been shown, that human CSN1S1 is expressed in human monocytes and induces pro-inflammatory cytokines [15]. Such ectopic expression of CSN1S1 could potentially be an explanation for the IgM mediated reaction on human CSN1S1 observed in the corresponding SD ELISA for a few sera. However, further systematic and thorough investigations will be required before such a relationship can be postulated.

It has been known for a long time that orally administered antigens can induce immune tolerance, when the same antigen was used in a further challenge. Therefore, oral tolerance has been employed for the treatment of human autoimmune diseases such as rheumatoid arthritis and multiple sclerosis [34]. In contrast, however, it was shown more recently that oral application of an autoantigen, in particular ovalbumin, can lead to a strong immune response in a corresponding mouse model [35] and finally was found to induce autoimmune diabetes [36]. The only general conclusion that can be drawn from summarizing these reports is that orally administered autoantigens can influence the peripheral immune system *via* the gut, either in a tolerance inducing or a stimulating manner. One could speculate about a possible role of human CSN1S1 in this context. Very recently we provided evidence that human CSN1S1 has an immunomodulatory function, that it is expressed by human monocytes and stimulates the expression of pro-inflammatory cytokine GM-CSF [8]. In the present study, we were able to show that human CSN1S1 induces a sustained IgG serum reaction in adults when applied orally during early infant development. This clearly indicates a task beyond its nutritional function in milk and could point on a more general role of human CSN1S1 during immune system development, e.g. in an early onset of the adaptive immune response, a hypothesis, of course, that needs further investigation.

The advantage of the SD ELISA presented here by application of the Autodisplay technology is that ELISAs for antibody-testing can be generated for a wide variety of antigens very rapidly and directly by expression on *E. coli*, Some disadvantage might be that - despite repeated stripping of the sera to be analyzed with control cells of *E. coli* – detectable amounts of antibodies directed against the host cell remain left. Therefore, serial dilutions in order to obtain exact antibody titers are not possible, and in addition, the control values obtained by analyzing the serum response against control cells of *E. coli* without antigen are usually exceeding zero. However, in such a scenario, it is very important to have a look on the absolute values obtained with controls cells and control, whether these values are significantly different (or not) from those obtained with cells displaying the antigen. In the present study, the mean of the values obtained with control cells in the breast fed consortium was 0.778, whereas the same mean in the formula fed consortium was 0.854. Both values turned out to be not significantly different. In contrast, the values obtained with CSN1S1 displaying cells in the breast fed consortium was 1.664, whereas the same mean in the formula fed consortium was 1.350. The means obtained with controls cells and with CSN1S1 displaying cells for both consortia were analyzed by one-way analysis of variance (ANOVA) with subsequent Sheffé-test using WinStat. version 2007.1 and it turned out, that both means in the breast fed consortium were significantly different, whereas in the formula fed group, both means were not significantly different (*P* = 0.001). This result was supported by applying a separate t-test for each serum in the breast fed consortium, comparing the mean values obtained with CSN1S1 displaying cells with those obtained with control cells. For all 35 sera, that showed a reaction beyond the cut off values of 0.4, the mean values were significantly different (*P* = 0.001), whereas for the sera, showing a reaction below the cut off value they were not significantly different.

In conclusion, we showed that there is an autoimmune reaction against human CSN1S1 and this autoimmune reaction in adults is orally determined by breast-feeding.

## Supporting Information

Figure S1 **Schematic description of the SD-ELISA for CSN1S1 detection in human sera.** (A) After induction of protein expression, cells of *E. coli* displaying the antigen – in the present study CSN1S1 – are used for coating the wells of a 96 well microplate overnight. After washing with PBS containing 0.1% tween, the sera to be tested on a reaction against the surface displayed antigen were applied for 1 h (B). Before application, the sera were stripped twice with cells of *E. coli* without the surface displayed antigen in order to remove the serum antibodies directed against the gram negative bacteria but not against CNS1N1. (C) After washing again with PBS containing 0.1% tween, a secondary antibody – goat anti-human IgG conjugated with horse radish peroxidase (HRP) is added. (D) The HRP substrate 3,3′,5,5′tetramethylbenzidine (TMB) is added and concentration of serum antibodies against CSN1S1 is quantified by measuring the absorption at 450 nm. For each serum to be analyzed three wells are treated identically and mean values and standard deviations are determined, as well as three wells are coated with *E. coli* without CSN1S1 and incubated with the same serum as control. The mean value obtained with the control was substracted from the mean value obtained with CSN1S1 displaying cells in order to minimize background absorption.
(PPT)

Figure S2 **Comparing the serum reaction against CSN1S1 of female and male test persons with respect to being breast-fed as a neonates.**
(PPT)

Figure S3 **Amino acid sequence alignment of bovine and human αS1-casein (CSN1S1) using the Smith and Waterman algorithm.** Amino acids (aa) are given in the one letter code. The region identified in 89% of patients suffering from cow milk allergy (CMA) by an overlapping dodecapeptide dot blot approach to be targeted by IgG antibodies (reference 31) is underlayed in green. Remarkably, the 93% identity observed between the bovine and the human protein within the first 15 aa belong to the signal peptide, which is supposed to be cleaved between a-l-a and r (between aa 15 and 16) after membrane transport.
(PPT)
