# Supplementary figures and images for: Autoantibodies to αS1-Casein Are Induced by Breast-Feeding
Source: PLoS One. 2012 Apr 4;7(4):e32716. doi: 10.1371/journal.pone.0032716 (PMC3319542; doi:10.1371/journal.pone.0032716)

## Slide 1
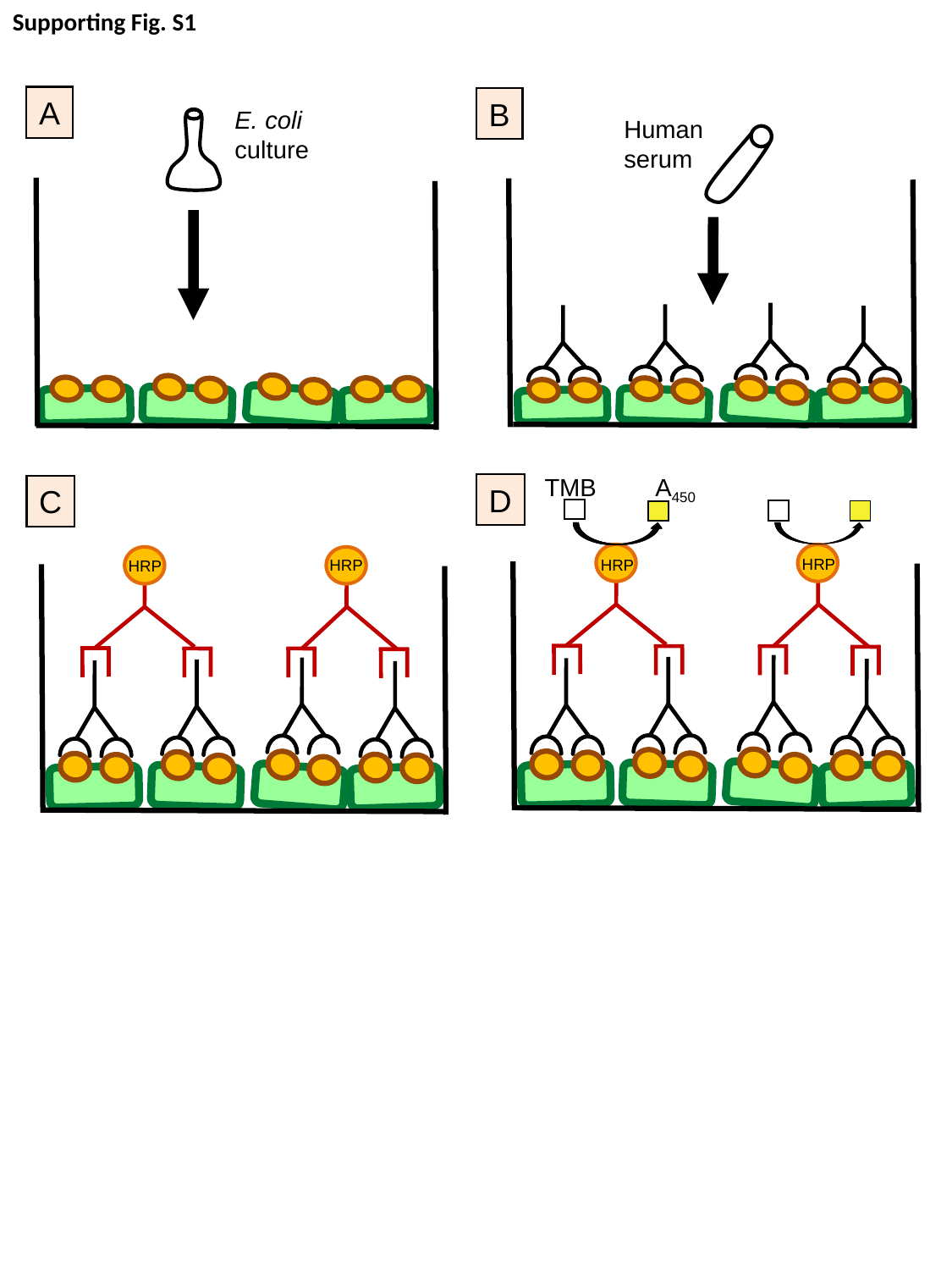

Supporting Fig. S1
A
B
E. coli
culture
Human
serum
TMB
A450
D
HRP
HRP
C
HRP
HRP

Supplement: Figure S1 — Schematic description of the SD-ELISA for CSN1S1 detection in human sera. (A) After induction of protein expression, cells of E. coli displaying the antigen – in the present study CSN1S1 – are used for coating the wells of a 96 well microplate overnight. After washing with PBS containing 0.1% tween, the sera to be tested on a reaction against the surface displayed antigen were applied for 1 h (B). Before application, the sera were stripped twice with cells of E. coli without the surface displayed antigen in order to remove the serum antibodies directed against the gram negative bacteria but not against CNS1N1. (C) After washing again with PBS containing 0.1% tween, a secondary antibody – goat anti-human IgG conjugated with horse radish peroxidase (HRP) is added. (D) The HRP substrate 3,3′,5,5′tetramethylbenzidine (TMB) is added and concentration of serum antibodies against CSN1S1 is quantified by measuring the absorption at 450 nm. For each serum to be analyzed three wells are treated identically and mean values and standard deviations are determined, as well as three wells are coated with E. coli without CSN1S1 and incubated with the same serum as control. The mean value obtained with the control was substracted from the mean value obtained with CSN1S1 displaying cells in order to minimize background absorption. (PPT) [file pone.0032716.s001.ppt]

## Slide 1
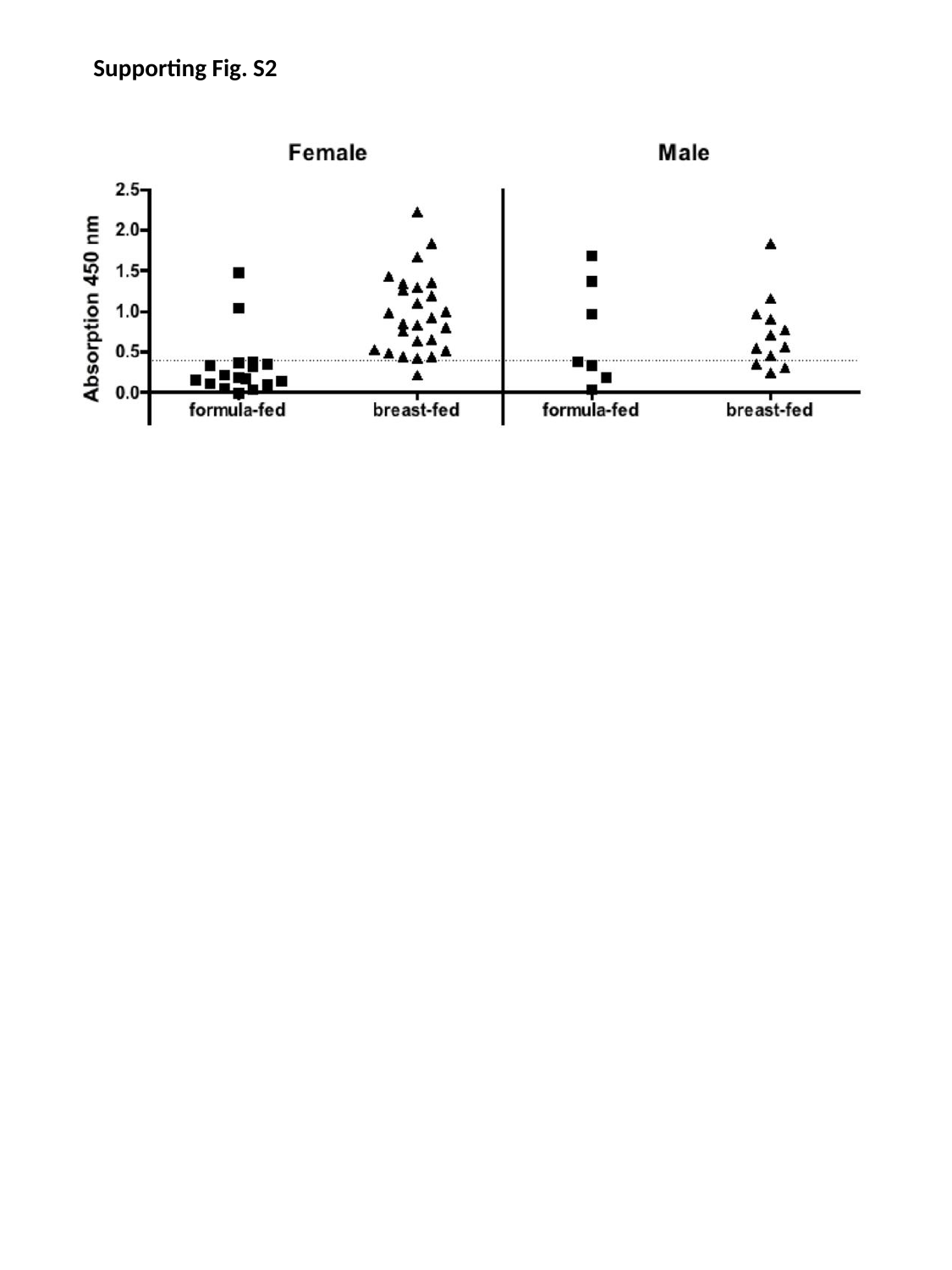

Supporting Fig. S2

Supplement: Figure S2 — Comparing the serum reaction against CSN1S1 of female and male test persons with respect to being breast-fed as a neonates. (PPT) [file pone.0032716.s002.ppt]
