# Supplementary material for: Autoantibodies to αS1-Casein Are Induced by Breast-Feeding
Source: PLoS One. 2012 Apr 4;7(4):e32716. doi: 10.1371/journal.pone.0032716 (PMC3319542; doi:10.1371/journal.pone.0032716)
Supplement: Figure S3 — Amino acid sequence alignment of bovine and human αS1-casein (CSN1S1) using the Smith and Waterman algorithm. Amino acids (aa) are given in the one letter code. The region identified in 89% of patients suffering from cow milk allergy (CMA) by an overlapping dodecapeptide dot blot approach to be targeted by IgG antibodies (reference 31) is underlayed in green. Remarkably, the 93% identity observed between the bovine and the human protein within the first 15 aa belong to the signal peptide, which is supposed to be cleaved between a-l-a and r (between aa 15 and 16) after membrane transport. (PPT) [file pone.0032716.s003.ppt]

## Slide 1
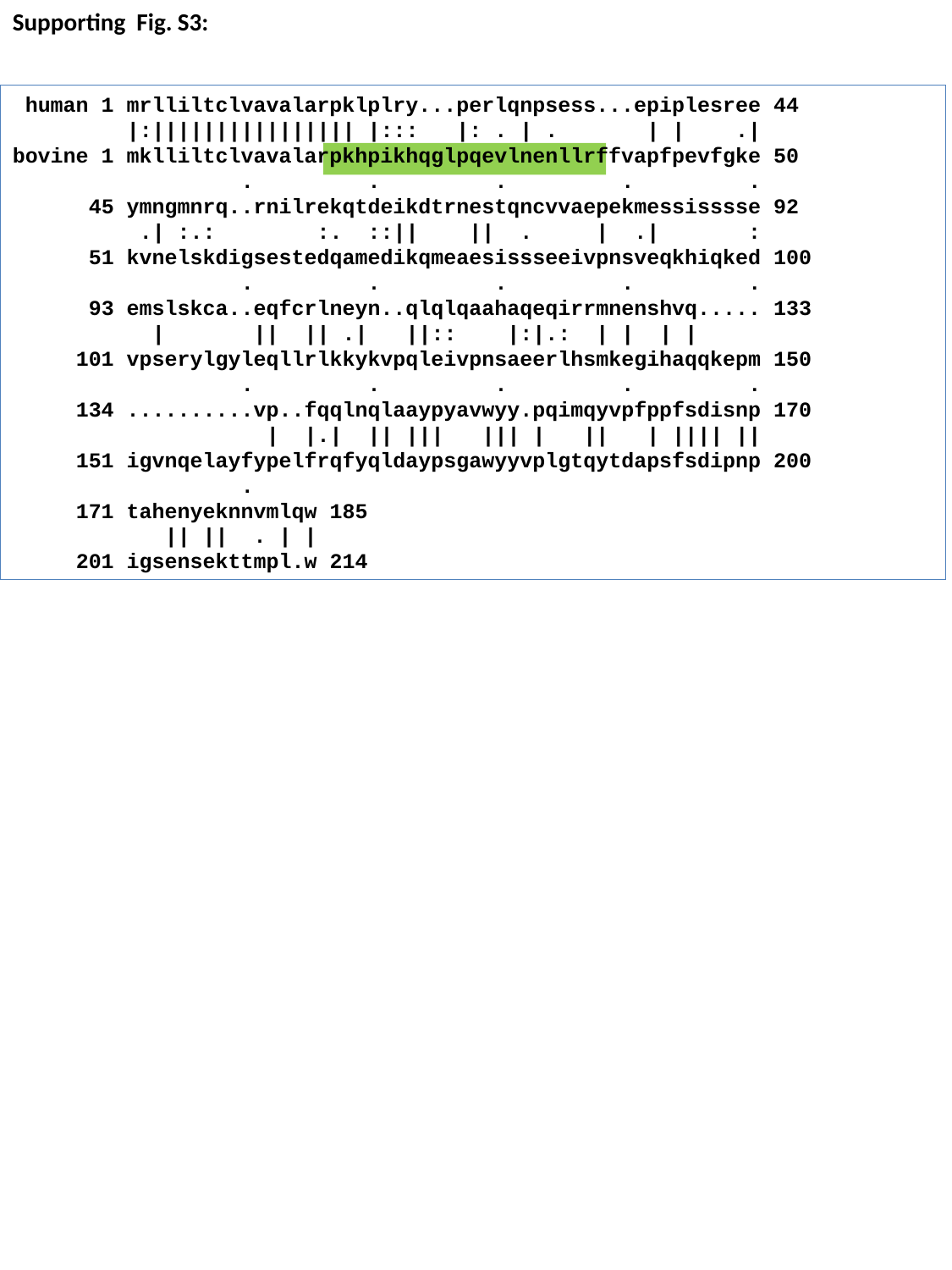

Supporting Fig. S3:
 human 1 mrlliltclvavalarpklplry...perlqnpsess...epiplesree 44
 |:|||||||||||||||| |::: |: . | . | | .|
bovine 1 mklliltclvavalarpkhpikhqglpqevlnenllrffvapfpevfgke 50
 . . . . .
 45 ymngmnrq..rnilrekqtdeikdtrnestqncvvaepekmessisssse 92
 .| :.: :. ::|| || . | .| :
 51 kvnelskdigsestedqamedikqmeaesissseeivpnsveqkhiqked 100
 . . . . .
 93 emslskca..eqfcrlneyn..qlqlqaahaqeqirrmnenshvq..... 133
 | || || .| ||:: |:|.: | | | |
 101 vpserylgyleqllrlkkykvpqleivpnsaeerlhsmkegihaqqkepm 150
 . . . . .
 134 ..........vp..fqqlnqlaaypyavwyy.pqimqyvpfppfsdisnp 170
 | |.| || ||| ||| | || | |||| ||
 151 igvnqelayfypelfrqfyqldaypsgawyyvplgtqytdapsfsdipnp 200
 .
 171 tahenyeknnvmlqw 185
 || || . | |
 201 igsensekttmpl.w 214
